# Supplementary material for: Biophilic classroom environments on stress and cognitive performance: A randomized crossover study in virtual reality (VR)
Source: PLoS One. 2023 Nov 1;18(11):e0291355. doi: 10.1371/journal.pone.0291355 (PMC10619869; doi:10.1371/journal.pone.0291355)
Supplement: S1 File — (A) Screening survey for participant recruitment which introduces the study, collects contact information, gender, age, ethnicity, and relevant health conditions. (B) Check-out survey which collects general health conditions, perceived stress levels, feelings of the connection with nature in the virtual classroom scenes, and preference for the three biophilic patterns. (DOCX) [file pone.0291355.s001.docx]

# S1A. Screening Survey

## Introduction

Please note that this round of experiment will take place at DKU campus during 2021 Fall semester. If you are not on campus currently, please stay tuned for the next round!

Below is the detailed introduction to the study, "The effect of biophilic classroom environment on stress reaction and cognitive function: a randomized crossover study in virtual reality (VR)."

Reading the following will take a maximum of 5 minutes.

**PURPOSE OF THE STUDY**

The purpose of this study is to examine the effect of biophilic classroom environment on stress reaction and cognitive function through a randomized crossover study in virtual reality (VR). Your participation in this experiment will help us understand whether biophilic elements like daylight, green plants, and natural materials as well as turbidity would visually affect people’s mood or be of help in reducing stress and relaxing people’s nerves.

**PARTICIPANTS SCREENING**

If you are interested, you will need to take an eligibility questionnaire. In this case, reading the following information and taking the screening questionnaire does not mean you are consenting.

If you are recruited to participate in the study, you can give your consent by signing a hard copy of consent form at the study site before the experienment starts.

**PROCEDURES**

The activity will take 65-83 minutes for one individual participant. After the introduction of the experiment and completion of the consent form, you will learn from the investigators about the general safety instructions and how to navigate the VR environment. The investigators will then help you wear the equipment. You will get familiar with the VR setup and conﬁrm that you indeed do not feel any discomfort.

After that, you will view the ﬁve synthetic environments in a random sequence.

For each scene:

Before viewing the picture, you will view a blank environment for rest and baseline measurement of blood pressure (BP). Then you will be exposed in a speciﬁc VR environment. After that, you will rate your current stress level on virtual desktop in VR, and the investigators will measure your BP by Omron J760, which would be placed on one of your arms. Heart rate (HR), heart rate variability (HRV), and skin conductance level (SCL) will be measured all the time. They will be measured by Shimmer GSR+Unit, which will be placed on your ﬁngers and an earlobe. Following you will complete two short cognitive tests called verbal backward digit span task and alternative use (AU) test. This would repeat for another four times for other scenes.

In the end, you will complete a short check-out survey. There would be questions asking your feelings of the VR scenes, whether you slept well last night, whether you drank caffeinated before the start of the experiment, whether you have myopia and the myopia degree, and your general health conditions.

The entire process will not be video-recorded. You will be audio-recorded during the alternative use test and the recording will be destroyed after the transcript has been made.

**POTENTIAL RISKS / DISCOMFORTS AND THEIR MINIMIZATION**

You may feel dizzy or discomfort wearing the VR device and seeing scenes through the VR headset. Please adjust your headset to the most comfortable position in the familiarization time. If at any point you want to seek support to mitigate any discomfort or confusion, please ask your investigators for help. You can also ask to stop the experiment any time if you feel uncomfortable continuing.

**COMPENSATION FOR PARTICIPATION**

You will be compensated with gifts valued at 50 RMB.

**POTENTIAL BENEFITS**

There is no direct beneﬁt for the participants.

However, we do hope that the participants will have a joyful experience on modern technologies such as virtual reality, and Bluetooth sphygmomanometer, etc.

We also hope the information and conclusions gained from this study will contribute the

research on exploring the impacts of biophilic elements on one’s stress level and will inspire the designers to decorate the room accordingly.

**CONFIDENTIALITY**

The research group promises strict conﬁdentiality and that the information obtained in the study will be used for research purposes only. Any personal data collected will not be used to deduce your identity as reporting data will only be a statistical summary of data analyses, and no identiﬁcation can be performed. All study results will be linked to study participants' ID number with no association to the names or other personal identiﬁers.

No video-recording of participants will take place.

**PARTICIPATION AND WITHDRAWAL**

Your participation is voluntary. This means that you can choose to stop at any time without negative consequences.

**QUESTIONS AND CONCERNS**

If you have any further questions about the research, feel free to contact Jicheng You at [jy260@duke.edu](mailto:jy260@duke.edu) or Xinyi Wen at [xw162@duke.edu](mailto:xw162@duke.edu).

## Questions

Would you like to sign up for the study?

Yes

No

To sign up, you need to take a short eligibility questionnaire (takes about 30 seconds). Do you want to continue?

Yes

No

### Demographic information

Your name

­­__________________

Your email

__________________

Your age

__________________

Your gender

Male

Female

Non-binary / third gender

Prefer not to say

### Health condition

Please check if you have any of the following health conditions.

Hypertension

Heart diseases

None of the above

Are you taking stress recovery medicine or participating in therapy related to stress relief?

Yes

No

### Results

Congratulations! You are eligible for this study! The study team will reach out to you within a week. Stay tuned :)

We are sorry to tell you that this study may not suit you. Thank you so much for your interest!

# S1B. Check Out Survey

## Demographic information

Your name

­­__________________

Your ethnicity

Asian

Non-Asian

Both

Prefer not to say

## Stress Level

Your current stress level is (1: very little stress; 5: extreme stress) -1

1 2 3 4 5

Your current stress level is (1: very little stress; 5: extreme stress) -2

1 2 3 4 5

Your current stress level is (1: very little stress; 5: extreme stress) -4

1 2 3 4 5

Your current stress level is (1: very little stress; 5: extreme stress) -5

1 2 3 4 5

## Health condition

Are you short-sighted?

No

Yes (please enter your diopter of myopia in the box below, e.g. L200, R200)

­­ __________________

Your general health condition is

Excellent

Very good

Good

Fair

Poor

Did you drink caffeinated beverage today or the day before?

Yes

No

Your sleep quality of the night before is

Excellent

Very Good

Good

Fair

Poor

## Feelings & Preferences

How much you feel the connection with nature in the four conditions you experienced just now (1: feel very little connection; 10: feel very strong connection)

0 1 2 3 4 5 6 7 8 9 10

Level of Connection: Non-biophilic classroom
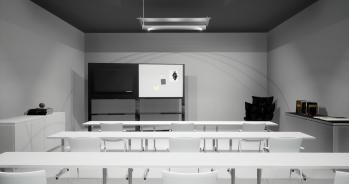


Level of Connection: Indoor green
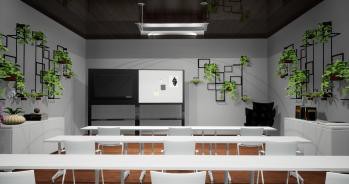


Level of Connection: Outdoor view
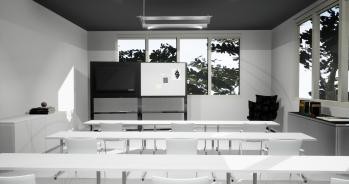


Level of Connection: Combination
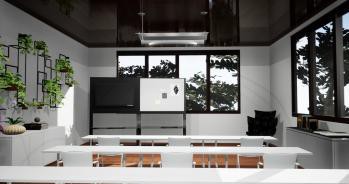


Level of Connection: Turbid Outdoor
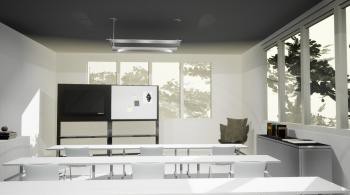


Please rank the following biophilic patterns in order of your preference

- Visual connection with nature (e.g., potted plants, windows with trees and sky)
- Dynamic & diffuse light (i.e., light and shadow)
- Material connection with nature (e.g., Wooden ﬂoor and ceiling)
