# Supplementary figures and images for: Biophilic classroom environments on stress and cognitive performance: A randomized crossover study in virtual reality (VR)
Source: PLoS One. 2023 Nov 1;18(11):e0291355. doi: 10.1371/journal.pone.0291355 (PMC10619869; doi:10.1371/journal.pone.0291355)

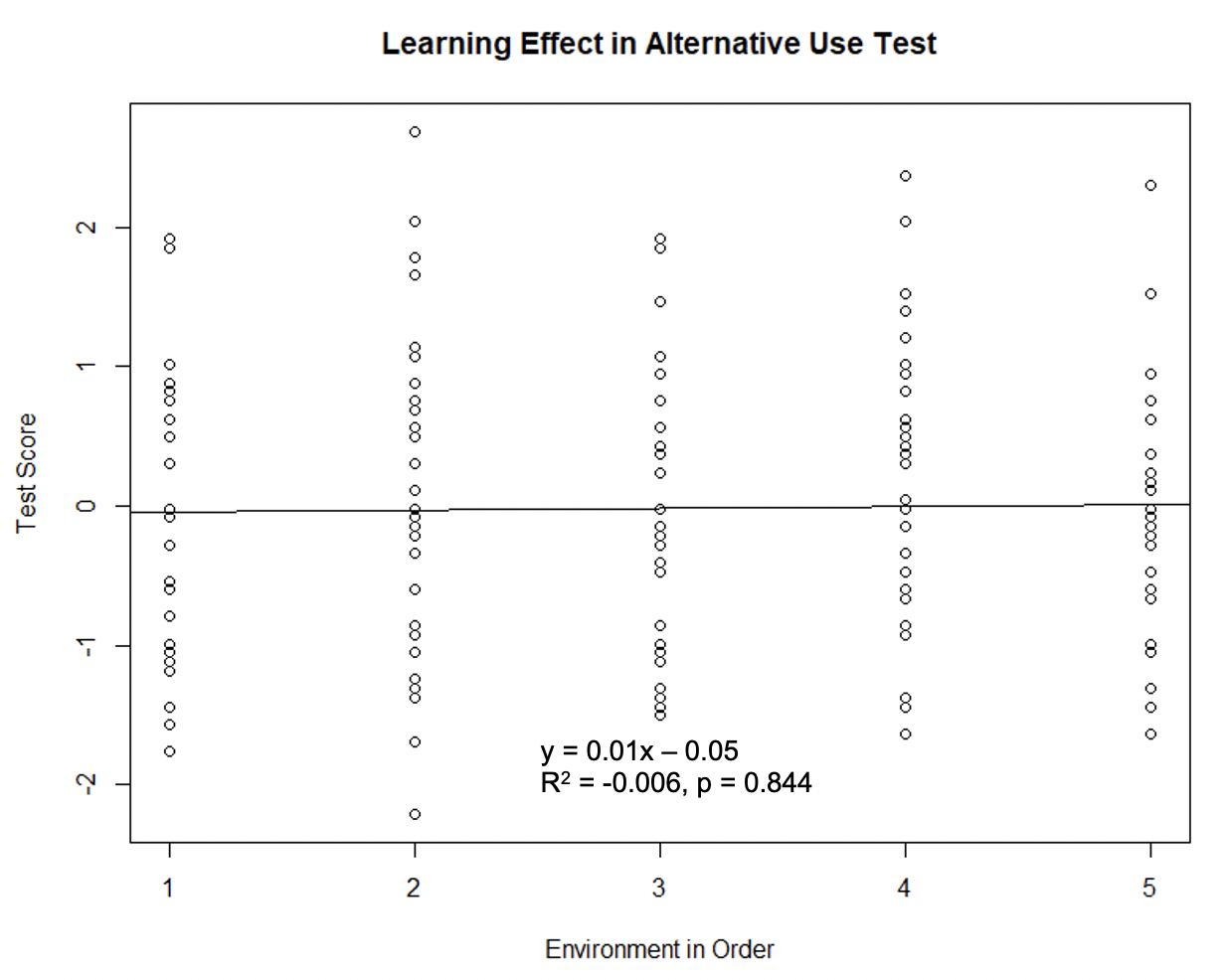

Supplement: S1 Fig — (A) Learning Effects in Verbal Backward Digit Span Task (Number test). With the adjustments of other variables, one more test was associated with 0.22 (95% CI: 0.051, 0.389) increase in the digit span. (B) Learning Effects in Alternative Use Test (AU test). No significant learning effect was detected in AU test. (ZIP) [file pone.0291355.s003.zip › S4/S4B Fig.tif]

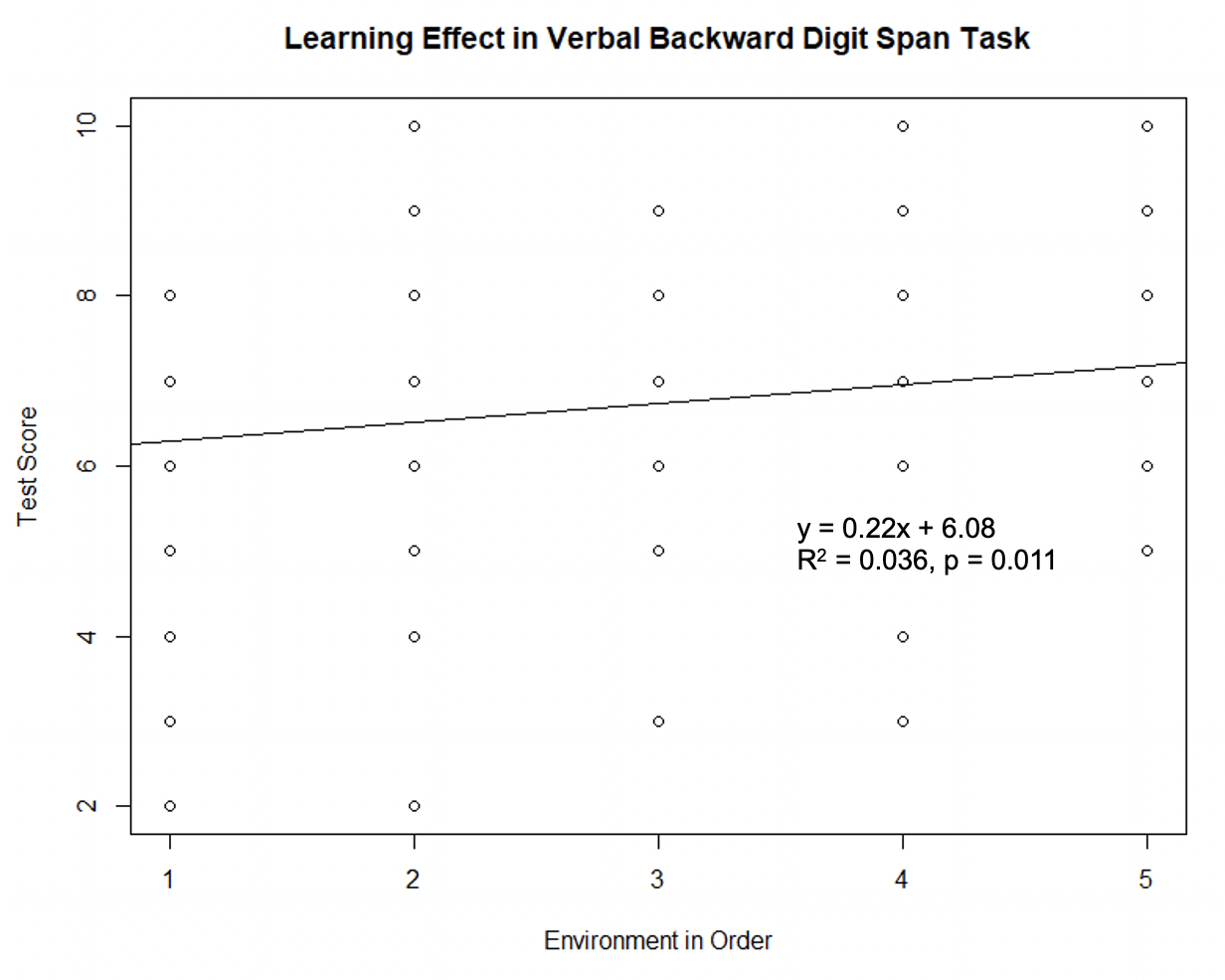

Supplement: S1 Fig — (A) Learning Effects in Verbal Backward Digit Span Task (Number test). With the adjustments of other variables, one more test was associated with 0.22 (95% CI: 0.051, 0.389) increase in the digit span. (B) Learning Effects in Alternative Use Test (AU test). No significant learning effect was detected in AU test. (ZIP) [file pone.0291355.s003.zip › S4/S4A Fig.tif]
