# Supplementary material for: Biophilic classroom environments on stress and cognitive performance: A randomized crossover study in virtual reality (VR)
Source: PLoS One. 2023 Nov 1;18(11):e0291355. doi: 10.1371/journal.pone.0291355 (PMC10619869; doi:10.1371/journal.pone.0291355)
Supplement: Attachment — Submitted filename: Revisione.docx [file pone.0291355.s006.docx]

**Review: Biophilic classroom environments on stress and cognitive performance: A randomized**

**a crossover study in virtual reality metaverse**

The current research investigated the effects of a virtual indoor environment with natural elements on cognitive functions and stress levels, based on the biophilia hypothesis. The authors conducted a randomized crossover study, proposing 30 Chinese students to four virtual classroom environments varying in biophilic elements and turbidity. They measured physiological indicators of stress (blood pressure, heart rate variability, and skin conductance level), as well as self-report measures of stress and connection with nature, and conducted attention and creativity tasks to assess cognitive functioning. Even though they did not find significant changes in stress reactions or cognitive functions, subjects showed a reduction in self-reported stress, and positive connections with nature were observed. The authors concluded that the addition of biophilic elements in the virtual environments could be beneficial to promote well-being.

This is very interesting research investigating possible new ways to exploit technological opportunities to promote human health. Some adjustments and clarifications might improve the work. I suggest authors consider the following comments and suggestions:

**Introduction:** It is not clear why you talked about “virtual reality Metaverse” instead of mere virtual reality. You might better explain this expression in the introduction;

**Introduction:** you might better present the current state-of-the-art about the use of virtual natural environments to promote well-being, focusing on a psychological perspective (e.g., Simone Grassini. 2022. The use of VR natural environments for the reduction of stress: an overview on current research and future prospective. In Proceedings of the 33rd European Conference on Cognitive Ergonomics (ECCE '22). Association for Computing Machinery, New York, NY, USA, Article 4, 1–5. <https://doi.org/10.1145/3552327.3552336>)

**Pag. 5 lines 101-102**: “The guiding role of visual senses in creating a perception” and “objective virtual exposure assessment” sound like abstract and unclear concepts. You might better explain these expressions (i.e., Do you refers to the dominance of the visual system in perception?).

**Pag. 6 line 105**: “short-term health” expression is not clear: what do you mean by short-term?

**Pag. 7 Environment Simulation section:** it is not completely clear why you choose to use indoor virtual environments since it is not fully exploited VR opportunities; I expected to find immersive naturalistic virtual environments instead of a classroom replication with some plants. Explain better your choice. You might also expand this aspect in the discussion/ future works.

**Pag. 7 Study Population**: were participants instructed to avoid smoking the day of the experiment or check for other possible intervening variables affecting physiological measures (excluding caffeine and subjective report of sleep quality)?

**Pag. 10 line 206**: did you use a VAS for the stress level rating? What was the item?

**Pag. 11 lines 217- 227**: Why did you choose attention and creativity? Are there previous studies specifically focused on these domains? You might better argue your choice. Additionally, in line 210 you stated the potential benefits of VR in boosting cognitive function assessment, but your paradigm tried to manipulate cognitive functions instead of assessing them.

**Pag. 11 line 218**: The science behind digit span reveals it's associated more with short-term memory. Why did you consider the Digit Span Test as an attentional task?

**Pag.12 line 248**: Why you did not include a questionnaire to assess the virtual experience (e.g., cybersickness, motion sickness)?

**Pag. 12 line 249**: Better explain the orientation test and the assumed effect on learning.

**Pag. 13 line 255**: Why 3 minutes?

**Pag. 21 Line 412**: Change the expression “biophilic intervention” (e.g., the exposure to different biophilic virtual scenarios)

**Pag. 22 Line 422- 425**: rephrase the sentence “we were able to partly confirm our hypothesis about the positive but different impacts of the biophilic interventions from the mental side, but could not confirm the negative impacts of the turbidity based on the current evidence” to make clearer your findings (e.g., in line with our hypothesis, the biophilic intervention was found to have positive effects in promoting subjective wellbeing).

**Pag. 22 “Stress reaction” section**: Why did you not consider emotional competence? There might be noticeable factors impacting the discrepancies between physiological and self-report measures. Given this is interesting data, it might be useful to briefly introduce this argument.

**Pag. 26 line 521-523**: “We used VR technology to bring participants to the Metaverse and explored their responses to the biophilic elements in this digital parallel universe connecting to the real world”. There are two issues in this sentence: first, as highlighted before it is not clear why you talk about Metaverse and not VR alone referring to the proposed experience, second, the “digital parallel universe” might sound a little bit exaggerated.

**Pag. 27 Line 543**: change “VR google”

**Pag. 27 “Limitations” sections**: Discuss better limitations, introducing also additional reflections (e.g., add the lack of a measure for cybersickness, possible variables affecting physiological activation such as time, smoking, hunger, the use of self-report measures and evaluations, gender differences).
