# Supplementary material for: Biophilic classroom environments on stress and cognitive performance: A randomized crossover study in virtual reality (VR)
Source: PLoS One. 2023 Nov 1;18(11):e0291355. doi: 10.1371/journal.pone.0291355 (PMC10619869; doi:10.1371/journal.pone.0291355)
Supplement: Attachment — Submitted filename: Response to Reviewers.docx [file pone.0291355.s008.docx]

1. **Line 90: the sentence “we used VR and wearable biomonitoring sensors to quantify the impacts of both positive and negative factors in built virtual environments on short-term health of university students” is not clear. What do the authors refer to by the term "short-term health"? Both stress and cognitive function? It would be better to clarify.**

The reviewer’s question for clarification is a clinically relevant point, as we should use more specific terminology. Short-term may mean different time scales according to different disciplines. In our language, we focused on non-chronic diseases, meaning that the etiology does not take many years to develop.

By “short-term health,” we mean immediate physiological and cognitive responses (i.e., physiological & perceived stress levels and cognitive tests performance) to the virtual scenes during the 90-minute experiment session. Furthermore, we also clarified “short-term” with added explanation that it refers to immediate impacts on health within the day, or min/hours following exposure (line 101-102, 115-116).

2. **It is suggested to add, in the introduction section, present studies in order to clarify what kind of link has been found in the literature between stress, creativity and exposure to natural environments (both real and virtual).**

The link between stress/cognitive function (including creativity) and exposure to **real** natural environments is discussed in the Introduction section as follows:

“Population epidemiology studies and experiments documented exposure to *outdoor* nature (e.g., greenspace) can positively affect human health and well-being in multiple ways: **reduced stress level, improved mental health and cognition**, lower mortality rates [12-16], and enhanced immune functions [17-20].” (line 70-74) and “There is emerging evidence that biophilic design in simulated environment can yield health benefits. For example, indoor plants could be conducive to **stress-reduction and attention restoration** [22, 23]” (line 81-83).

The link between stress/cognitive function (including creativity) and exposure to **virtual** natural environments is discussed in the Introduction section as follows:

“Combined biophilic design elements using virtual reality (VR) by Yin et al. [30] demonstrated that bringing nature into virtual indoor workspace has clear benefits to the health outcomes, including physiological **stress reductions and cognitive function (attention and creativity) improvements**.” (line 88-92).

In terms of the link between stress and creativity, we did not address that in the manuscript since it is not part of our research question.

3. **Reviewer 1 noticed that Verbal Backward Digit Span Task (Number test) is a test for working memory and not for attention; Authors responded citing literature that conflates the two constructs. This is satisfying; however, it seems that nothing has been changed in the manuscript in this regard. Authors should add this specification and the related citation to the manuscript, not only in Response to Reviewers.**

We revised the manuscript accordingly and added the citation (line 238-239). We may have had an oversight changing many aspects in the previous version. We thank the reviewer for their fastidiousness in ensuring our manuscript is scientifically sound with respect to test and corresponding cognitive domains.

4. **We recommend adding in the discussion or limit section studies that support the role of interaction with the virtual environment in stress reduction (see, for example, Liszio, S., & Masuch, M. (2019). Interactive immersive virtual environments cause relaxation and enhance resistance to acute stress. Annu Rev Cyberther Telemed, 17, 65-719). In fact, it may be possible that participants' lack of interaction with your study material reduced the possibility of achieving the expected results regarding stress reduction through other measurements as well.**

Absolutely, and it is essential that we address the limitations appropriately. As you know, our study participants were mainly students, and this population is healthy but also has many virtual inputs throughout the day. Certainly, if there are more interactions in the virtual space, it may change the dynamic of the biological response measurements. To capture this point, we added this argument in the Strengths and Limitations section as a recommendation for future VR studies (line 642-645).

5. **Line 465: "After removing the anomalies, no significant differences were found for physical measurements of physiological indicators." Also emphasize here that no differences were found at the cognitive level.**

We emphasized this point and revised it accordingly (line 483). Although, we think our finding can instigate more studies as better digital biomarkers technologies become available. At this point, subjective finding is a start, and the lack of physiological response finding may be due to ability to detect differences, or because of sample size. We thank the reviewer in making sure that this point comes across properly.
